# Pure estrogen receptor antagonists potentiate capecitabine activity in *ESR1-*mutant breast cancer

**DOI:** 10.1038/s41523-024-00647-1

**Published:** 2024-06-08

**Authors:** Albert Grinshpun, Douglas Russo, Wen Ma, Ana Verma, Francisco Hermida-Prado, Shira Sherman, Giorgio Gaglia, Sheheryar Kabraji, Gregory Kirkner, Melissa E. Hughes, Nancy U. Lin, Zachary Sandusky, Agostina Nardone, Cristina Guarducci, Quang-De Nguyen, Sandro Santagata, Zsuzsanna Nagy, Rinath Jeselsohn

**Affiliations:** 1 Susan F. Smith Center for Women’s Cancers, Dana-Farber Cancer Institute, Boston, MA USA; 2 Department of Medical Oncology, Dana-Farber Cancer Institute, Boston, MA USA; 3 Harvard Medical School, Boston, MA USA; 4 Department of Data Science, Dana-Farber Cancer Institute, Boston, MA USA; 5 Ludwig Center at Harvard, Harvard Medical School, Boston, MA USA; 6 Department of Pathology, Brigham and Women’s Hospital, Harvard Medical School, Boston, MA USA; 7 Lurie Family Imaging Center, Center for Biomedical Imaging in Oncology, Dana-Farber Cancer Institute, Boston, MA USA; 8 Center for Functional Cancer Epigenetics, Dana-Farber Cancer Institute, Boston, MA USA

**Keywords:** Breast cancer, Breast cancer

## Abstract

The *ESR1* ligand binding domain activating mutations are the most prevalent genetic mechanism of acquired endocrine resistance in metastatic hormone receptor-positive breast cancer. These mutations confer endocrine resistance that remains estrogen receptor (ER) dependent. We hypothesized that in the presence of the ER mutations, continued ER blockade with endocrine therapies that target mutant ER is essential for tumor suppression even with chemotherapy treatment. Here, we conducted comprehensive pre-clinical in vitro and in vivo experiments testing the efficacy of adding fulvestrant to fluorouracil (5FU) and the 5FU pro-drug, capecitabine, in models of wild-type (WT) and mutant ER. Our findings revealed that while this combination had an additive effect in the presence of WT-ER, in the presence of the Y537S ER mutation there was synergy. Notably, these effects were not seen with the combination of 5FU and selective estrogen receptor modulators, such as tamoxifen, or in the absence of intact P53. Likewise, in a patient-derived xenograft (PDX) harboring a Y537S ER mutation the addition of fulvestrant to capecitabine potentiated tumor suppression. Moreover, multiplex immunofluorescence revealed that this effect was due to decreased cell proliferation in all cells expressing ER and was not dependent on the degree of ER expression. Taken together, these results support the clinical investigation of the combination of ER antagonists with capecitabine in patients with metastatic hormone receptor-positive breast cancer who have experienced progression on endocrine therapy and targeted therapies, particularly in the presence of an *ESR1* activating mutation.

## Introduction

Hormone receptor-positive (HR + ) breast cancer (BC) accounts for the majority of primary and metastatic BC worldwide^1^. Endocrine therapy (ET) is the mainstay therapy in early and advanced estrogen receptor-positive (ER + ) BC. Chemotherapy is also an important component of adjuvant systemic treatment in patients with high risk early-stage ER + BC and in the management of metastatic disease, especially after patients have exhausted ET and other targeted therapy options^1^.

Previous studies investigated the combination of chemotherapy and ET in metastatic BC^2^. These studies have yielded mixed results, likely due to several limitations, such as inclusion of patients with hormone receptor-negative (HR-) BC and the utilization of older generation chemotherapy and ET regimens. The SWOG-8814 phase III trial tested the combination of chemotherapy and tamoxifen in early-stage HR + BC. In this study, post-menopausal women with lymph node-positive disease were randomized to adjuvant treatment with tamoxifen alone, chemotherapy followed by tamoxifen or chemotherapy given concurrently with tamoxifen^3^. Although, the 10-year disease free-survival and overall survival were numerically lower in the patients that received tamoxifen concurrently with chemotherapy compared to sequentially, the difference did not reach statistical significance^3^. Overall, these clinical trials did not support the combination of chemotherapy and tamoxifen and currently the combination of ET and chemotherapy is not standard of care.

Despite the clinical data opposing the combination of tamoxifen and chemotherapy, there is clinical evidence to suggest that other classes of ET, such as the pure estrogen receptor (ER) antagonist and selective estrogen receptor degrader (SERD), fulvestrant, may enhance the efficacy of chemotherapy. In a phase II trial of patients with HR+ metastatic BC who received fulvestrant in combination with capecitabine the median progression-free survival was 15 months^4^, compared to historical data showing a median progression-free survival of ~7 months with capecitabine alone^5^.

Published pre-clinical studies provide mechanistic insights to support the combination of chemotherapy and SERDs. Several studies showed an interaction between ER and P53^6–9^, with ER activation repressing P53 induced transcription of pro-apoptotic genes in response to chemotherapy^8,9^. Moreover, tamoxifen displayed agonistic activity in combination with chemotherapy and blocked the transcription of P53 induced pro-apoptotic genes. In contrast, fulvestrant de-repressed P53 mediated transcription and enhanced the response to chemotherapy^9^. Collectively, these studies suggest that ER blockade with a pure ER antagonist could enhance the efficacy of chemotherapy.

In recent years the ER ligand binding domain (LBD) activating mutations have emerged as the most common genetic mechanism of resistance to aromatase inhibitors in metastatic HR + BC. These mutations lead to constitutive activity and are a mechanism of resistance to ET in which ER signaling remains active but altered. Moreover, we showed that these mutations possess neomorphic properties that promote metastases, and silencing of these mutations can lead to significant regression of metastases in a xenograft model, indicating oncogenic addiction to these mutations in the metastatic setting^10^. Importantly, these mutations are prognostic of decreased overall survival in metastatic HR + BC and recently the next-generation ET, elacestrant, was approved for the treatment of ER-mutant metastatic BC^11,12^. Therefore, we hypothesized that continued blockade of the ER mutations even after initiating chemotherapy in metastatic HR + BC could improve clinical outcomes. To test this hypothesis and evaluate the anti-tumor effects of combining chemotherapy and a SERD in wild-type (WT) and mutant ER, we conducted a comprehensive analysis of the combination of chemotherapy and fulvestrant in in vitro and in vivo pre-clinical models. Given the wide use and relatively good tolerance of the oral chemotherapy capecitabine in metastatic HR + BC coupled with evidence supporting enhanced clinical benefit from the combination of capecitabine and fulvestrant, our studies were focused on capecitabine, or its active form 5FU, in combination with fulvestrant.

## Results

### Fulvestrant combined with chemotherapy has additive to synergistic activity in ER+ breast cancer cells

**Fig. 1:**
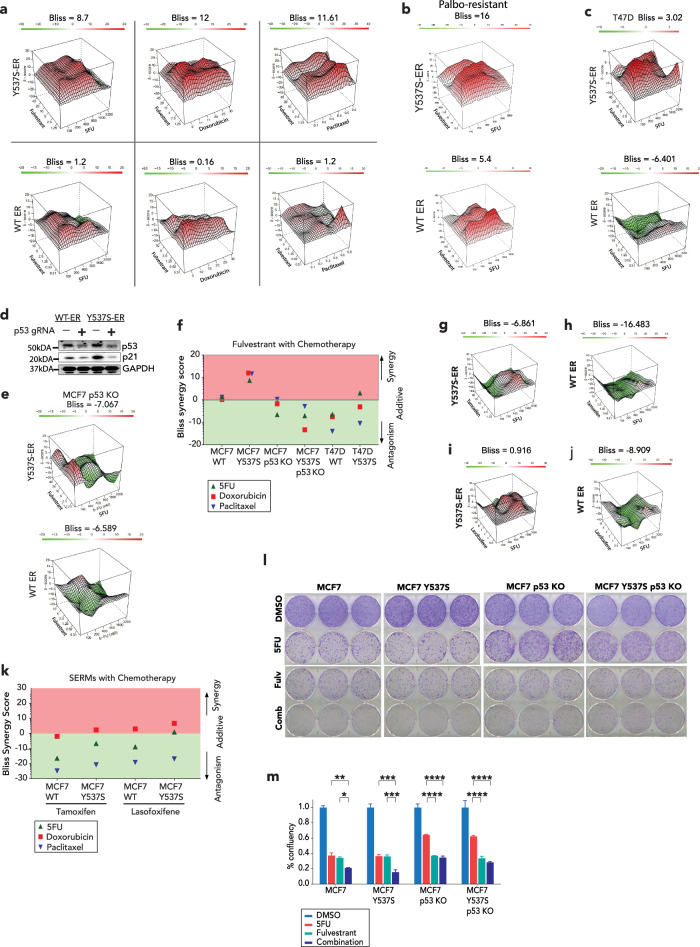
Combination of fulvestrant and chemotherapy have synergistic effects in ER+ breast cancer. **a**–**c** Drug combination plots with Bliss scores of MCF7 cells expressing either WT ER (lower panels) or the doxycycline induced Y537S ER mutation (upper panels) (**a**), treated with fulvestrant in combination with 5FU, doxorubicin or paclitaxel. **b** Drug combination plots with Bliss scores performed of palbociclib-resistant MCF7 cells expressing either the induction of the Y537S mutation (upper panel) or WT ER (lower panel), treated with fulvestrant in combination with 5FU. **c** Drug combination plots with Bliss scores of *TP53* mutant T47D cells expressing the doxycycline induced Y537S mutation or WT ER treated with fulvestrant in combination with 5FU. **d** Immunoblot analysis of whole cell lysates for p53 and p21 expressions in control or p53 knock-out MCF7 cells expressing either WT ER or the doxycycline induced Y537S mutation. The blots were derived from the same experiment and were processed in parallel. **e** Drug combination plots with Bliss scores of P53 knock-out MCF7 cells expressing the doxycycline induced Y537S mutation or WT ER, treated with fulvestrant in combination with 5FU. **f** Summary of Bliss synergy scores for each cell line representing fulvestrant treatment in combination with various chemotherapies. **g**, **h** Synergy studies with Bliss scores of MCF7 cells expressing either doxycycline induced Y537S mutation (**g**) or WT-ER (**h**), treated with tamoxifen in combination with 5FU. **i**, **j** Drug combination plots with Bliss scores of MCF7 cells expressing either the doxycycline induced Y537S mutation (**i**) or WT ER (**j**) treated with tamoxifen in combination with 5FU. **k** Summary of Bliss synergy scores for each cell line representing tamoxifen or lasofoxifene treatment in combination with various chemotherapies. **l**, **m** Colony confluency of control or *TP53* knock-out MCF7 cells expressing WT ER or the inducible Y537S mutation, following treatment with fulvestrant either as single agent or in combination with 5FU. Bliss scores: >10 denotes synergy; <−10 denotes antagonism and between −10 to 10 denotes an additive effect (https://synergyfinder.fimm.fi/synergy/synfin_docs/). The drug doses are shown in nMol. Data are shown as mean ± SE. Two-way ANOVA testing was used for comparison with Tukey’s multiple comparison test for (**m**). *P < 0.05, **P < 0.01, ***P < 0.001, ****P < 0.001. SERM selective estrogen receptor modulator, ER estrogen receptor, WT wild-type, KO knock-out.

To investigate the impact of the Y537S mutation on response to chemotherapy, we utilized the MCF7 and T47D cell lines engineered to stably express the Y537S ER mutation upon doxycycline (DOX) induction. First, we assessed the IC50 values of three commonly used chemotherapy agents (5FU, doxorubicin and paclitaxel) in MCF7 and T47D cells without and with DOX induction of the Y537S ER mutation (Supplementary Fig. 1a, b). For all three chemotherapy agents and in both cell lines (except for doxorubicin in MCF7 cells) we observed a mild increase in the IC50 values following the DOX induced expression of the Y537S ER mutation (Supplementary Fig. 1a, b), indicating partial resistance to chemotherapy engendered by the Y537S ER mutation. Consequently, we examined the impact of adding fulvestrant, an ER antagonist and SERD, to chemotherapy (5FU, doxorubicin and paclitaxel) through a series of drug combination studies. Remarkably, in MCF7 cells expressing the Y537S ER mutation, Bliss scores indicated synergistic or near synergistic activity with the three chemotherapy agents (Fig. 1a, upper panels). In contrast, MCF7 cells without the mutant ER consistently showed an additive effect (Fig. 1a, lower panels). Notably, the combination of 5FU and fulvestrant was highly synergistic in MCF7 cells that acquired resistance to palbociclib in the presence of the Y537S mutation. Additive activity was seen in the palbociclib-resistant cells expressing WT-ER (Fig. 1b).

Since previous studies have shown that ER represses P53-mediated transcription, we hypothesized that the additive or synergistic activity of fulvestrant in combination with chemotherapy might be, at least in part, P53-dependent^9^. This was confirmed in T47D cells harboring an L194F *TP53* mutation, in which the combination of fulvestrant and chemotherapy resulted in lower Bliss scores compared to MCF7 cells, regardless of the ER mutational status (Fig. 1c). Furthermore, silencing P53 in MCF7 cells resulted in decreased Bliss scores and abolished the synergistic activity of fulvestrant in combination with chemotherapy, irrespective of the presence of the Y537S ER mutation (Fig. 1d–f).

A previous study demonstrated that tamoxifen, a selective estrogen receptor modulator (SERM), functions as an ER agonist when in combination with chemotherapy and represses P53 mediated transcription^9^. In keeping with this observation, the combination of tamoxifen (Fig. 1g) or a second SERM, lasofoxifene, with 5FU had lower Bliss scores compared to the combination of chemotherapy with fulvestrant with evidence of antagonistic activity when tamoxifen is combined with chemotherapy in cells with WT-ER (Fig. 1g–k). Collectively, these findings suggest that the addition of a SERD to chemotherapy enhances anti-tumor activity, especially in the presence of the Y537S mutation. Furthermore, this additive to synergistic effect is in part P53 dependent and is not seen with a SERM.

To functionally test the cell growth effects of combining fulvestrant with chemotherapy, we performed colony formation experiments. We focused these studies on 5FU, since the oral 5FU pro-drug, capecitabine, is widely used as the first-line chemotherapy treatment in metastatic HR+ after patients develop resistance to ET-based regimens. Consistent with the drug combination studies, the confluency was significantly decreased with the treatment combination compared to 5FU or fulvestrant alone in the presence of WT and mutant ER. In addition, P53 silencing reduced the efficacy of 5FU activity and in this context, the addition of fulvestrant to 5FU did not enhance tumor growth suppression compared to fulvestrant alone (Fig. 1l, m and Supplementary Fig. 1c).

### Fulvestrant inhibits ER-mutant mediated transcription and potentiates the suppression of cell proliferation when combined to 5FU

**Fig. 2:**
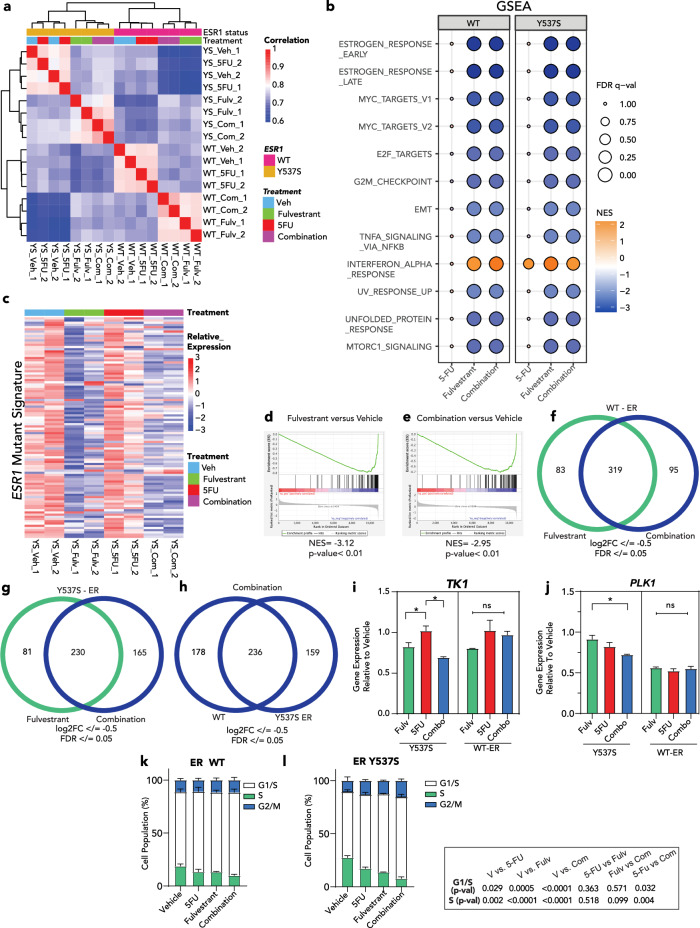
Combination therapy effects are mediated through cell cycle inhibition in vitro. **a** Sample-sample Spearman correlations of cells with or without expression of the Y537S mutation treated with vehicle (Veh), 5FU, fulvestrant (Fulv), or 5FU in combination with fulvestrant (Com). Correlations were calculated using log2(FPKM + 1) gene expression values among the top 1000 most variable genes. Rows and columns are hierarchically clustered using Euclidean distance among correlations and Ward’s squared linkage. **b** Normalized enrichment scores (NES) and FDR *q*-values from GSEA-Pre-ranked run on MCF7 cells and Hallmark gene sets for log2FC-ranked gene lists from testing contrasts of 5-FU, Fulvestrant, and Combination to vehicle treated cells for WT and Y537S mutant samples respectively. Gene sets that had an FDR *q*-value < 0.01 for at least one of the six contrasts are shown. **c** *ESR1* mutant gene signature for MCF7 cells expressing the inducible Y537S mutation treated with vehicle (Veh), 5FU, fulvestrant (Fulv), or 5FU in combination with fulvestrant (Com) showing relative expression values across samples (row centered and scaled log2(FPKM + 1) expressions) with rows and columns unclustered. *ESR1* mutant gene signature consists of the top 100 genes ranked by the log2 fold change *−log10(adjusted pvalue) that are upregulated in Y537S mutant vehicle treated cells compared to WT vehicle treated cells (Supplementary Data 1e). **d**, **e** GSEA Pre-ranked enrichment plots for the mutant *ESR1* gene signature for MCF7 cells expressing the Y537S mutation run on DESeq2 contrasts of fulvestrant (**d**) and combination (**e**) compared to vehicle treated controls showing normalized enrichment scores (NES) and their permutation *P* values. **f**, **g** Venn diagrams showing the number of genes downregulated in MCF7 cells without (**f**) or with (**g**) the expression of Y537S mutation for fulvestrant (Fulv) and 5FU in combination with fulvestrant (Com) compared to vehicle treated cells for log2FC <= −0.5 and FDR < = 0.05 filtering thresholds. **h** Venn diagram showing the number of genes downregulated in MCF7 cells treated with 5FU in combination with Fulvestrant (Com) compared to vehicle treated cells between cells without (WT) or with (Y537S ER) expression of the Y537S mutant for log2FC <= −0.5 and FDR <=0.05 filtering thresholds. **i**, **j** Gene expression of *TK1* (**i**) and *PLK1* (**j**) in MCF7 cells with or without the expression of the Y537S ER mutation treated with vehicle (Veh), 5FU, fulvestrant (Fulv) or 5FU in combination with fulvestrant (Combo). **k**, **l** Cell cycle analysis of MCF7 cells without (**k**) or with (**l**) the expression of the Y537S mutation treated with vehicle (V), 5FU, fulvestrant (Fulv) or 5FU in combination with fulvestrant (Com). Data are shown as mean ± SE. Two-way ANOVA testing with Tukey’s multiple comparison test was used for comparisons for (**i**–**l**). *P < 0.05. ER estrogen receptor, GSEA gene set enrichment analysis, ns non-significant, FC fold change, FDR false discovery rate.

To gain molecular insights to the additive and synergistic activity of fulvestrant and 5FU in Y537S ER-mutant and WT-ER BC cells, respectively, we analyzed early transcriptomic changes in MCF7 cells. MCF7 cells with and without expression of the Y537S ER mutation were treated with vehicle control, fulvestrant, 5FU and fulvestrant plus 5FU for 12 h. Sample to sample correlation analysis grouped the cells based on the expression of the Y537S ER mutation (Fig. 2a). Within these two clusters (WT and mutant ER), cells treated with fulvestrant and fulvestrant plus 5FU separated from the cells treated with 5FU or vehicle. Differential gene expression analysis (DESeq2, |log2FC | >0.5, FDR < 0.05) revealed that, at this relatively early time point, there were no significant gene expression changes after 5FU treatment in either WT or Y537S mutant cells, consistent with the clustering of the vehicle and 5FU treated cells. Conversely, fulvestrant and the combination of fulvestrant plus 5FU significantly downregulated 402 and 414 genes, respectively, and upregulated 195 and 205 genes, respectively in WT-ER cells (Supplementary Fig. 2 and Supplementary Data 1a–d).

Hallmark pathway analysis using Gene Set Enrichment Analysis (GSEA), revealed that genes downregulated with fulvestrant and the combination of fulvestrant plus 5FU were enriched in genes related to ER signaling, MYC signaling, the cell cycle (E2F [G0/G1] and G2/M), and mTOR signaling (Fig. 2b). To test the impact of the different treatments specifically on ER-mutant mediated transcription, we created a custom ER-mutant gene set comprised of the top 100 genes upregulated in vehicle treated MCF7 cells after the DOX induction of the Y537S ER mutation (Supplementary Data 1e). This gene set included several genes that have key roles in metastases and inflammation such as SOX9^13,14^ and S100P^15,16^. As shown in Fig. 2c, treatment with fulvestrant alone and fulvestrant plus 5FU, but not 5FU alone, resulted in lower expression of these genes compared to vehicle treatment. The effect of fulvestrant and the combination of fulvestrant plus 5FU on the ER-mutant gene set was validated by GSEA (Fig. 2d, e). As expected, the downregulation of the ER-mutant gene set was not seen in ER-WT cells (Supplementary Fig. 3a).

Although the Hallmark GSEA analysis of differentially expressed genes after fulvestrant and fulvestrant plus 5FU in WT and Y537S mutant ER cells was similar, a comparison at the gene level, revealed unique gene expression changes in each treatment condition (Fig. 2f, g and Supplementary Fig. 3b, c). Furthermore, the genes that were differentially expressed after the combination treatment versus vehicle control did not completely overlap when comparing WT-ER cells with Y537S mutant ER (Fig. 2h and Supplementary Fig. 3d). Genes with key functions in the regulation of cell proliferation, such as TK1 and PLK1, were among those uniquely downregulated after the combination treatment in the presence of the Y537S mutation (Fig. 2i, j). Lastly, cell cycle analysis showed that the addition of fulvestrant to 5FU significantly increased the accumulation of cells in the G1/S phase and decreased the percentage of cells in the S-phase in cells expressing the Y537S mutant ER (Fig. 2k, l), supporting the RNAseq findings. Together, these results suggest that the addition of fulvestrant to 5FU suppresses ER signaling and the cell cycle and these effects are likely potentiated through the inhibition of ER-mutant mediated signaling and enhanced cell cycle inhibition in the presence of an ER mutation.

### Fulvestrant augments the response to capecitabine in an ER-mutant PDX model

**Fig. 3:**
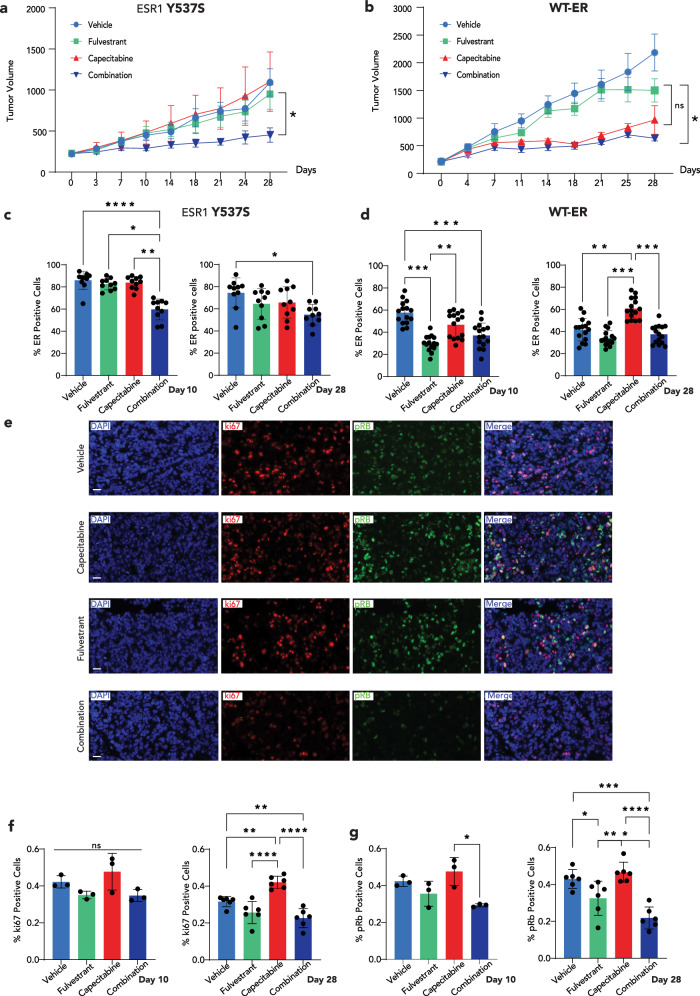
Combination therapy suppresses in vivo tumor growth in mutated ER setting. **a**, **b** Tumor volumes over 28 days of treatment with vehicle, fulvestrant, capecitabine or capecitabine in combination with fulvestrant in a *TP53* WT PDX model expressing **a** Y537S mutated ER or **b** WT ER. *N* = 5–8 mice per group. The data represents mean tumor volume (mm^3^) and error bars denote ± SE. **c**, **d** Percentage of ER positive cells (by immunohistochemistry) in PDX tumors harvested at 10 or 28 days following treatment with vehicle, fulvestrant, capecitabine or capecitabine in combination with fulvestrant and expressing Y537S mutated ER (**c**) or WT ER (**d**). **e** Representative immunofluorescence images for DAPI, Ki67 and Rb phosphorylated at Serines 807/811 (p-Rb) staining from PDX models expressing Y537S mutated ER and treated with vehicle, fulvestrant, capecitabine or capecitabine in combination with fulvestrant for 28 days. Scale bar = 20 micron. **f** Percentage of Ki67 positive cells in the Y537S ER mutant PDX following 10 days (*n* = 3) or 28 days (*n* = 6) of treatment with vehicle, fulvestrant, capecitabine or capecitabine in combination with fulvestrant. **g** Percentage of p-Rb positive cells in the Y537S ER mutant PDX following 10 days (*n* = 3) or 28 days (*n* = 6) of treatment with vehicle, fulvestrant, capecitabine or capecitabine in combination with fulvestrant. Data are shown as mean ± SE. Two-tailed Mann–Whitney test was used for comparison of tumor volumes. Kruskal–Wallis test was used for tissue staining comparisons. *P < 0.05. **P < 0.01, ***P < 0.001, ****P < 0.0001. PDX patient-derived xenograft, WT wild-type, ER estrogen receptor, ns non-significant.

We next investigated the in vivo impact of combining fulvestrant to capecitabine employing two patient-derived xenograft (PDX) models of HR+ (ER + /PR + /HER2−) BC. These PDX models were derived from metastatic BC lesions, with one harboring a Y537S ER mutation (1526) and the other with WT-ER (1415). The mice were treated with vehicle, fulvestrant, capecitabine, or the combination of fulvestrant plus capecitabine for 28 days, and additional mice were treated for 10 days to assess the molecular effects at an early timepoint. In the Y537S mutant model, neither fulvestrant nor capecitabine alone significantly inhibited tumor growth. However, the combination of fulvestrant plus capecitabine resulted in a significant reduction in tumor growth at day 28 (Fig. 3a). The WT-ER PDX model was resistant to fulvestrant, but sensitive to capecitabine alone or the combination of fulvestrant and capecitabine, but in this model combining fulvestrant with capecitabine did not impact tumor growth significantly (Fig. 3b). Interestingly, in the Y537S ER-mutant model the ER expression decreased only after treatment with the combination of fulvestrant and capecitabine (Fig. 3c). In contrast, in the WT-ER model, ER expression decreased with fulvestrant alone and remained suppressed after the combination of fulvestrant and capecitabine (Fig. 3d). Overall, these results suggest that in this WT-ER model that is resistant to fulvestrant, ER suppression combined with capecitabine treatment did not augment tumor inhibition but also did not compromise the effect of capecitabine.

To further validate the impact of adding fulvestrant to capecitabine in the Y537S ER- mutant PDX model, we assessed the levels of Ki67 and pRB. The addition of fulvestrant to capecitabine resulted in a time dependent decrease in both markers when compared to capecitabine alone, with significant changes observed after 28 days of treatment (Fig. 3e–g).

**Fig. 4:**
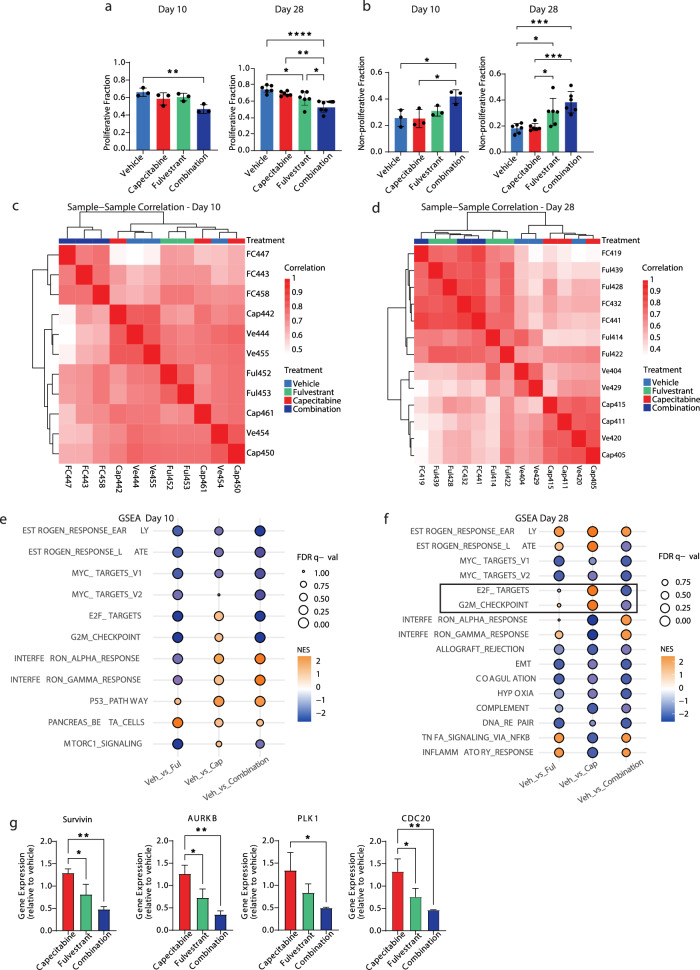
Combination therapy suppresses ER transcriptional activity in vivo. Multi-parametric immunofluorescence analysis (MPI) of proliferative (**a**), and non-proliferative (**b**), epithelial tumor cells from the PDX tumors expressing the Y537S ER mutation and treated with vehicle, fulvestrant, capecitabine or capecitabine in combination with fulvestrant for 10 days or 28 days. **c**, **d** Sample-sample Spearman correlations of PDX tumors expressing the Y537S mutation treated with vehicle (Ve), fulvestrant (Ful), capecitabine (Cap), or capecitabine in combination with fulvestrant (FC) harvested at day 10 (**c**) and day 28 (**d**). Correlations were calculated using log2(FPKM + 1) gene expression values among the top 1000 most variable genes. Rows and columns are hierarchically clustered using Euclidean distance among correlations and Ward’s squared linkage. **e**, **f** Normalized enrichment scores (NES) and FDR *q*-values from GSEA-Pre-ranked run on PDX tumors expressing the Y537S mutation with Hallmark gene sets for log2FC-ranked gene lists from testing contrasts of fulvestrant (Fulv), capecitabine (Cap), or capecitabine in combination with fulvestrant (FC) compared to vehicle (Veh) at day 10 (**e**) and day 28 (**f**). Gene sets that had an FDR *q*-value < 0.01 for at least one of the three contrasts in each panel are shown. The black rectangle highlights the specific effects of combination therapy on cell cycle-related pathways after 28 days of therapy. **g** Gene expression of *Survivin, AURKB, PLK1* and *CDC20* in PDX tumors expressing the Y537S ER mutation and treated with various agents. Staining data represent mean ± SEM. Plots show average gene expression ± SEM (*n* = 3–4 mice/group). Kruskal–Wallis test was used for tissue staining comparisons. *P < 0.05, **P < 0.01, ***P < 0.001, ****P < 0.0001. PDX patient-derived xenograft, GSEA gene set enrichment analysis, FDR false discovery rate.

Our in vitro and in vivo studies demonstrated that the addition of fulvestrant to 5FU repressed the expression of genes associated with the cell cycle and proliferation. Therefore, we conducted a comprehensive cell proliferation analysis using the Multivariate Proliferation Index (MPI)^17^. The MPI incorporates multiplex immunofluorescence (IF) staining intensities of three proliferation markers (Ki67, PCNA, MCM2) and two markers of cell-cycle arrest (p21, p27). MPI classifies each cell as either proliferative, if the cell expresses a positive balance of the proliferation markers, non-proliferative for a cell that lacks the expression of proliferative markers, and arrested if the cell expresses one or two of the cell-cycle arrest markers, irrespective of the expression of the proliferation markers. In the Y537S mutant PDX, most of the cells in the vehicle treatment arm were classified as proliferative (Fig. 4a, b). Already at day 10, there was a significant decrease in the fraction of the proliferative cells with fulvestrant plus capecitabine, but not with fulvestrant or capecitabine alone (Fig. 4a). Correspondingly, the cell fraction of non-proliferation of cells increased when fulvestrant was combined with capecitabine (Fig. 4b). By day 28, fulvestrant treatment alone resulted in a significant reduction of the proliferative fraction, and the combination of fulvestrant with capecitabine led to superior suppression of the proliferative fraction compared to each drug alone (Fig. 4a). Although, the addition of fulvestrant to capecitabine reduced the fraction of arrested cells at day 10 compared to capecitabine alone, there was no significant difference in the fraction of arrested cells comparing these two treatment groups by day 28 (Supplementary Fig. 4a). Overall, the fraction of the arrested cells was marginal and therefore changes in this fraction are limited and likely not of biological significance.

To gain a broader understanding of the molecular effects resulting from the addition of fulvestrant to capecitabine, we performed transcriptomic analyses of the tumors harvested at day 10 and 28 from all treatment groups. At day 10, sample to sample clustering showed that the tumors treated with fulvestrant combined with capecitabine were distinct from the other tumors (Fig. 4c). By day 28, the combination and fulvestrant treated tumors clustered together and segregated from the capecitabine alone and vehicle control tumors, aligning with the results observed in the cell lines (Fig. 4d).

Differential gene expression testing revealed that the number of genes that were significantly up or downregulated compared to vehicle control increased at day 28 compared to day 10 in all treatment arms (Supplementary Fig. 4b, c). While capecitabine alone had a relatively limited impact on gene expression at both time points, the addition of fulvestrant to capecitabine resulted in significant differential expression (up plus down) of 858 genes at day 10 and 3758 genes at day 28 (|log2FC | >0.5, FDR < 0.01). Pathway analysis highlighted key differences between the treatment arms and between day 10 and day 28 (Fig. 4e, f). At day 10, genes related to pathways of estrogen response (early and late) and the cell cycle (G0/G1 [E2F targets] and G2/M) were downregulated with fulvestrant monotherapy and the combination of fulvestrant and capecitabine but not with capecitabine alone. In addition, P53 pathway genes were upregulated with capecitabine alone and the combination of capecitabine and fulvestrant. Strikingly, by day 28, cell cycle-related genes and ER responsive genes (late) were no longer suppressed by fulvestrant or capecitabine alone, suggesting resistance to treatment in the residual tumors. In contrast, the combination treatment continued to suppress ER (estrogen response late) signaling and cell cycle associated genes. Furthermore, at day 28 the expression of the inhibitor of apoptotic gene, *survivin*, and key cell cycle genes, such as *AURKB*, *PLK1* and *CDC20*, was significantly lower with fulvestrant plus capecitabine compared to each drug alone (Fig. 4g).

### The addition of fulvestrant to capecitabine decreases proliferation in ER-high and ER− low cells

**Fig. 5:**
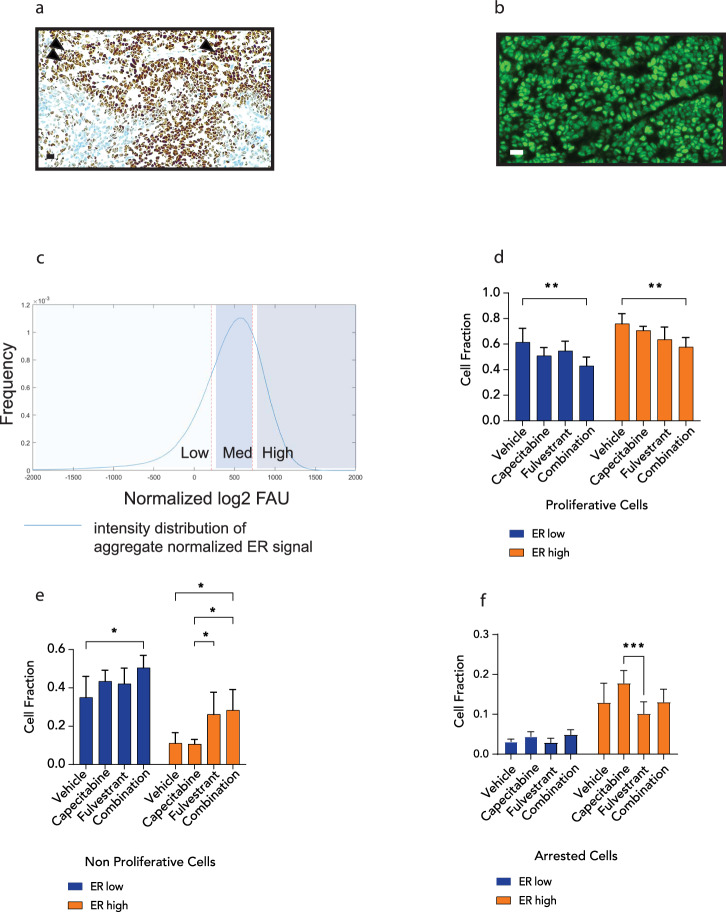
Combination therapy decreases proliferation of breast cancer cells in vivo regardless of ER expression. Representative immunohistochemistry (**a**) and immunofluorescence (**b**) images of ER staining in PDX tumors expressing the Y537S ER mutation following 28 days of vehicle treatment. Single arrowhead points to cells with intense ER expression, while the two black arrowheads show cells with lower expression of ER. Scale bar = 20 micron. **c** Normalized ER expression of epithelial tumor cells from Y537S ER PDXs at 28 days of treatment. Classifications include low, medium and high ER expression. Number of proliferative (**d**), non-proliferative (**e**), and arrested (**f**) cells in low vs. high ER expression groups from PDX tumors expressing the Y537S ER mutation and treated with vehicle, fulvestrant, capecitabine or capecitabine in combination with fulvestrant. Data represents mean ± SE. One-way ANOVA testing was used for comparisons. Two-way ANOVA was used to compare within the ER low and ER high groups. *P < 0.05. **P < 0.01, ***P < 0.001. PDX patient-derived xenograft.

Since BC exhibits cellular heterogeneity in ER expression (Fig. 5a, b), we categorized the BC cells from our PDX study as ER-low and ER-high and analyzed the impact of fulvestrant, capecitabine and the combination on these two sub-populations (Fig. 5c). When considering overall proliferation with MPI, we observed a reduction in the proliferative cells and a corresponding increase in the non-proliferative cells with the combination treatment compared to vehicle control in both ER-high and ER-low cells (Fig. 5d, e). Thus, these results indicate that the benefit from the addition of fulvestrant to capecitabine is observed in all cells and is most likely due to the additive to synergistic activity of the combination of these drugs within single cells rather than selective inhibition of the high ER expressing cells by fulvestrant and the low ER expressing cells by capecitabine.

## Discussion

The treatment in HR+ metastatic BC involves a sequential approach usually commencing with targeted therapies. This includes the use of ET in combination with CDK4/6 inhibitors, followed by the combination of ET and agents targeting the PI3Kinase-AKT-MTOR pathway, and eventually transitioning to chemotherapy regimens. Recently antibody drug conjugates (ADC), such as sacituzumab govitecan and trastuzumab deruxtecan^18^, have gained approval for the treatment of metastatic ER + BC. However, despite evidence for ER-dependent mechanisms of resistance to ET, such as the ER LBD activating mutations, ET is discontinued when patients switch to chemotherapy or ADC regimens.

In our study, we investigated the combination of fulvestrant, a pure ER antagonist and ER degrader, in combination with 5FU and the oral pro-drug capecitabine in pre-clinical models with WT and the Y537S mutation. Our findings demonstrated that in the presence of an ER mutation, the addition of ET to 5FU has a synergistic effect in vitro and an additive effect in vivo, resulting in increased suppression of cell proliferation and tumor growth, respectively. Notably, when fulvestrant was added to capecitabine in the absence of an ER mutation, the impact was modest, however, importantly, this combination did not diminish the effects of 5FU or capecitabine, nor was it antagonistic. These results indicate that the G1/S arrest induced by ET does not compromise the efficacy of chemotherapy even though chemotherapy primarily targets the S and M phases of the cell cycle. Of significant clinical relevance, our study aligns with the encouraging results from a single-arm clinical trial investigating the combination of capecitabine and fulvestrant^4^.

In this study, we demonstrated that the ER mutations potentiate the additive activity of a SERD in combination with chemotherapy leading to synergistic activity. The in vitro and in vivo PDX studies provide molecular insights to explain this effect. Firstly, we observed that 5FU alone does not inhibit the expression of genes induced by the expression of the ER mutations, whereas the addition of fulvestrant effectively suppressed these genes. Secondly, we showed that the addition of fulvestrant to 5FU enhanced the cell cycle suppression and this effect persisted even after long-term treatment. In the PDX models, by day 28, RNAseq results demonstrated the loss of cell cycle suppression with fulvestrant or capecitabine alone, whereas, with the addition of fulvestrant to capecitabine, cell cycle-related genes remained suppressed. Additionally, we demonstrated that the additive to synergistic activity is, at least in part, dependent on P53 activity. In the context of 5FU treatment, this effect may be attributed to the key functions of P53 in regulating the cell cycle through the regulation of p21. Importantly, since mutual exclusivity between *ESR1* and *TP53* mutation was detected in metastatic tissue biopsies, metastatic lesions harboring an *ESR1* mutation may not have a mutant P53^19^. It is also noteworthy that P53 activity is likely more critical when combining a SERD with a chemotherapy agent such as doxorubicin, which induces DNA damage and P53 dependent cell death.

A previous pre-clinical study demonstrated that the combination tamoxifen, a partial ER antagonist that has context dependent agonistic activity, with adriamycin resulted in the repression of P53 mediated transcription of pro-apoptotic genes in a manner similar to estrogen stimulation, suggestive of agonistic activity of tamoxifen in this context^9^. Similarly, we did not observe additive or synergistic activity when 5FU was combined with a SERM, suggesting that in this setting the agonistic activity of tamoxifen may be the predominant effect. Therefore, we hypothesize that the combination of chemotherapy and tamoxifen will not improve patient outcomes. This is supported by the clinical trials testing combinations of chemotherapy with tamoxifen^2^. In our pre-clinical models, we investigated the combination of fulvestrant and 5FU. Although the ER mutations confer relative resistance to fulvestrant as a single agent, as previously observed in pre-clinical and clinical studies^12,20^, in vitro resistance can be circumvented by using higher concentrations of fulvestrant, as we have used in the in vitro synergy studies. However, for clinical investigation, a next-generation oral SERD with improved bioavailability and efficacy in the presence of an *ESR1* mutation^21^, would likely be a better ET partner.

Collectively, our study warrants clinical investigation of the combination of a next-generation oral SERD with capecitabine in metastatic ER + BC, particularly in patients with cancers harboring an *ESR1* mutation. Furthermore, we demonstrated that in addition to 5FU, other chemotherapies such as paclitaxel and doxorubicin exhibit additive to synergistic activity in combination with a SERD in tumors with an *ESR1* mutation, suggesting the potential clinical benefit of continuing ER blockade in metastatic BC even after switching to second- or third- line chemotherapy or an antibody drug conjugate, such as trastuzumab-deruxtecan and sacituzumab-govitecan.

## Methods

### Cell lines

MCF7 and T47D breast cancer cell lines were obtained from the American Type Culture Collection (ATCC) (Manassas, VA, USA). MCF7 and T47D cells were maintained in Dulbecco’s modified Eagle’s medium (DMEM) and Roswell Park Memorial Institute (RPMI) respectively, supplemented with 10% fetal bovine serum (FBS) and 10 µg/ml penicillin-streptomycin-glutamine (PSG).

Palbo-resistant models were developed by culturing the cells with increasing concentration of palbociclib, from 50 nM to 1 µM. Cells were defined as resistant to palbociclib when they restore the ability to proliferate in the presence of palbociclib 1 µM. Palbociclib-resistant MCF7 cells were cultured in DMEM supplemented with 10% FBS, 10 µg/ml PSG and palbociclib 1 µM.

MCF7 cells with a doxycycline-inducible Y537S mutation (MCF7-Y537S) in ER and T47D cells with a doxycycline-inducible Y537S mutation (T47D-Y537S) were created as previously described^10^. Briefly, the Y537S mutant *ESR1* cDNA was cloned into a pInducer20 plasmid (Addgene, #44012). The plasmid was packaged into viral particles in HEK293T cells, using Lenti-XTM HTX Packaging System (Clontech, #631247 and #631249). MCF7-Y537S and T47D-Y537S cells were cultured in DMEM and RPMI respectively, supplemented with 10% FBS, PSG and 500 µg/ml of Geneticin. MCF7-Y537S or T47D-Y537S cells were treated with 1 µg/ml doxycycline for three days to induce the expression of the mutation.

MCF7-Y537S cells with TP53 knock-out were previously published^22^. Briefly, TP53 knock-out was done by lentiviral transduction using the lentiCRISPRv2 (Addgene #89567) transfer vector and pMD2.G (Addgene; #12259) and psPAX (Addgene; #12259) lentiviral packaging vectors. The following single guide (sg)RNA sequences were used: sgTP53

Forward: CATTGTTCAATATCGTCCG; sgTP53 Reverse: CATGGGCGGCATGAACCGG.

MCF7-Y537S cells with TP53 knock-out were cultured in DMEM supplemented with 10% FBS, 10 µg/ml PSG and 1 mg/mL puromycin.

All cell lines used were authenticated by short tandem repeat profiling (Bio-Synthesis, USA) free from mycoplasma, and cultured at 37 °C/5% CO_2_ in a humidifier incubator.

### Western blotting

Cell pellets were lysed with RIPA lysis buffer (Boston BioProducts) freshly supplemented with protease inhibitor cocktail (Sigma-Aldrich). Protein lysate was standardized using the BCA protein quantitation assay kit as per manufacturer’s instructions (ThermoFisher Scientific, Waltham, MA, USA), and 20–40 μg whole protein lysates were resolved on either 10% or 4–12% NuPage Bis-Tris gels (Life Technologies) and blotted on Trans-Blot® Turbo™ Midi Nitrocellulose membranes (Bio-Rad Laboratories). Membranes were blocked with 5% (wt/vol) BSA in Tris-buffered saline with Tween-20 (20 mM Tris-HCl (pH 7.6), 137 mM NaCl, 0.1% Tween-20), then incubated overnight at 4 °C with the following primary antibodies: p53 (1:1000, DO-1; Sigma-Aldrich); p21 (1:1000, 12D1; Cell Signaling Technologies) and GAPDH (D16H11; Cell Signaling Technologies). Appropriate horseradish peroxidase-conjugated secondary antibodies (1:2500; Santa Cruz Biotechnologies) were diluted in Tris-buffered saline with 0.1% Tween-20 and membranes were probed at room temperature for 2 h. Immunoblots were visualized with Pico ECL Chemiluminescence reagents (ThermoFisher Scientific). Figures of the gel and molecular weight markers are in Supplementary Fig. 5.

### Synergy studies and IC50 assays

Cells were plated in 96-well plates at 10,000 cells/well density. After 24 h, cells were treated with the following drugs and serial doses based on the calculation of IC50’s in MCF7 cells with and without the expression of the Y537S ER mutation: tamoxifen (Sigma-Aldrich) for WT-ESR1: 0.3, 0.6, 1.2, 2.5, 5, 10 nM and Y537S mutant ESR1 1.2, 2.5, 5, 10, 20, 40 nM; for WT *ESR1* condition fulvestrant (Selleckchem) in 0.3, 0.6, 1.2, 2.5, 5, 10 nM, and for *ESR1* Y537S-expressing cells in 1.2, 2.5, 5, 10, 20, 40 nM; lasofoxifene (Santa Cruz) in 0.5, 1, 2, 4, 8, 16 nM; doxorubicin (Abmol Bioscience) in 5, 10, 15, 20, 25, 30 nM; 5-fluorouracil (5FU) (Selleckchem) in 100, 200, 400, 800, 1600, 3200 nM; and paclitaxel (Selleckchem) in 0.1, 0.2, 0.3, 0.4, 0.5, 0.6 nM. On day 5, cells were counted using the Celigo image Cytometer (Nexcelom Bioscience, Lawrence, MA). Hoechst (0.4 µg/ml) was used for nuclear staining and propidium iodide (4 µg/ml) was used to stain dead cells. For the IC50 studies, the number of live cells after drug treatments was normalized to the number of cells treated with DMSO at day 5 and the IC50 values were calculated in Graphpad Prism using the log(inhibitor) vs. response – Variable slope (four parameters) function. Synergy (bliss score) was calculated using the SynergyFinder website (https://synergyfinder.fimm.fi/synergy/20231114183415308177/).

### Colony formation

MCF7, MCF7-Y537S and MCF7-Y537S TP53 knock out cells were seeded in 6-cm^2^ plates at 2000 cells/well density and treated with vehicle, 5FU (800 nM), fulvestrant (40 nM) or fulvestrant in combination with 5FU for 14 days, to allow colonies to form. MCF7-Y537S and MCF7-Y537S *TP53* knock out cells were kept under doxycycline (1 µg/ml) treatment for the duration of the study. Colonies were fixed and stained with 0.1% crystal violet solution (Sigma-Aldrich) then washed with water to remove the unincorporated stain. Cells were photographed and the confluency of the colonies was quantified using ImageJ software (https://imagej.nih.gov/ij/download.html).

### Cell cycle analysis

Cells were seeded into 96-well plates at 3000 cells/well density. The plated cells were starved in FBS-free culturing medium for 16 h for cell cycle synchronization, followed by treatment with vehicle, fulvestrant, 5FU or fulvestrant in combination with 5FU. After 24 h of drug exposure, cell cycle assay was performed using the Click-iT™ EdU Alexa Fluor™ 488 HCS Assay (ThermoFisher Scientific) according to the manufacturer’s instructions. Newly synthesized DNA was labeled with EdU (5-ethynyl-2’-deoxyuridine), positive cells were counted, and the cell cycle phases were estimated using the Celigo image Cytometer.

### Patient-derived xenografts

All mice were maintained in accordance with local guidelines and therapeutic interventions approved by the Animal Care and Use Committees of Dana-Farber Cancer Institute. These PDX were published previously^10^. For patient-derived xenograft (PDX) studies, patient consent for tumor implantation in nude mice was obtained under an IRB approved protocol (Dana-Farber/Harvard Cancer Center IRB protocol 93-085) and with patient consent and in compliance with the Declaration of Helsinki. Tumor samples from the ER-WT PDX1415 and the Y537S ER-mutant PDX1526 were dipped in 50% matrigel and implanted into the fourth mammary fat pads of ovariectomized NOD-SCID-IL2Rgc–/– mice (Jackson Laboratories) without estradiol (E2) supplements for the 1526 model and supplemented with 0.18 mg (60 days release) E2 pellets for the PDX1415. For the Cell injections and E2 pellet implantation the mice were anaesthetized by inhalation of isoflurane mixed with medical air. When tumors reached 150–200 mm^3^, mice were randomized into 4 arms (*N* = 5–8): control (drug vehicle), fulvestrant 5 mg/mouse/week subcutaneous, capecitabine 150 mg/kg/daily oral and fulvestrant 5 mg/mouse + capecitabine 150 mg/kg. Capecitabine was administered in a cycle consisting of 14 days on, 7 days off and again 7 days on. Fulvestrant vehicle was 10% ethanol and castor oil, while capecitabine vehicle was 10% Dimethylsulfoxide (DMSO), 40% polyethylene glycol, 2.5% Tween-80, and 47.5% normal saline. Tumor volume was measured at least once a week. After treatment mice were euthanized, and tumors were harvested for molecular biological studies. Mouse euthanasia was performed by exposure with carbon dioxide (CO2), followed by cervical dislocation. For each mouse, a half of the tumor was snapped frozen for DNA and RNA extraction and the other half was fixed in formalin and paraffin embedded (FFPE) for immunohistochemistry staining.

### Tissue staining

Immunohistochemistry was performed on the Leica Bond III automated staining platform using the Leica Biosystems Refine Detection Kit. Antibody against ER (Fisher/Neomarkers, catalog number: RM-9101-S1, clone SP1) was run at 1:40 dilution with citrate antigen retrieval. Images at ×20 magnification were taken using the Leica DM750 Microscope and ICC50 Leica Camera. Quantification was performed using the QuPath image analysis software (https://qupath.readthedocs.io/en/0.4/docs/intro/citing.html).

### Cyclic immunofluorescence (CyCIF)

FFPE slides were baked at 60 °C for 30 min, dewaxed using Bond Dewax solution at 72 °C, and antigen retrieval was performed with Epitope Retrieval 1 solution at 100 °C for 20 min using the BOND RX Automated IHC/ISH Stainer. Antibodies for each cycle were diluted in Odyssey Blocking Buffer and incubated overnight at 4 °C in the dark (used antibody list in Supplementary Data 2). After antibody incubation, slides were stained with Hoechst 33342 for 10 min at room temperature. Slides were cover slipped using 20–50% glycerol solution (Sigma, G5516) in PBS. Images were taken using DAPI, FITC, Cy3, and Cy5 channels either on the RareCyte CyteFinder (20×/0.75NA objective). After imaging, fluorophores were inactivated (4.5% H_2_O_2_, 20 mM NaOH in PBS, 45 min) under LED lights, and the next cycle was performed.

CyCIF image processing is organized in the following steps detailed further below. Additional details and access to the underlying code can be found at https://github.com/labsyspharm/ashlar and https://github.com/santagatalab. Stitching, registration, correction of acquisition artifacts were performed using ASHLAR and the BaSiC algorithm. Ilastik software was trained on cropped images to label nuclear, cytoplasmic, and background areas. Pan-cytokeratin positivity was used as the threshold for determining epithelial cells, which are the cells which were included in the analysis. Data aggregation, filtering, normalization and analysis were performed as previously described^17^. Multivariate Proliferation Index (MPI) calculation was published previously^17^. Briefly, MPI is based on the normalized measurement of 5 markers: three proliferation markers (Ki-67, MCM2, PCNA) and two cell cycle arrest markers (p21, p27). The method avoids relying on single markers while separating cells expressing high level arrest markers (even if proliferation markers are expressed). The determination of threshold values for proliferation and arrest is dataset dependent. For ER high vs. low analysis, entire cohort was used to divide ER expression into four quartiles. The top and bottom quartiles were addressed as ER high and low, respectively.

### RNA sequencing

MCF7 cells without (WT-ER) or with DOX induced expression of the Y537S ER mutation were treated with vehicle (DMSO), 5FU (1000 nM), fulvestrant (10 nM), or combination treatment for 12 h. Then, cells were harvested (as duplicates) for downstream RNA analysis. Total RNA was extracted using the QIAGEN RNeasy kit with DNase digestion. RNA concentrations were measured by NanoDrop, and the quality of RNA was determined by a Bioanalyzer. RNA-seq libraries were made using the TruSeq RNA Sample Preparation Kit (Illumina). Samples were sequenced on an Illumina Nextseq500. The VIPER pipeline was used for alignment, quality control, and downstream analysis^23^. Alignment to the hg19 human genome was done using STAR v2.7.0 f followed by transcript assembly using cufflinks v2.2.1. Quality control steps were done using STAR v2.7.0 f and RseQC v2.6.4. We assessed each sample on metrics of mappable reads, percentage of rRNA reads, gene body coverage, and junction saturation and insert size for paired end to determine samples that were of adequate quality. Differential expression testing was done using DESeq2 v1.22.1 and plots were created with R v4.3.1 using ggplot2 v3.4.4 and pheatmap v1.0.12^24–27^. Sample-sample correlation heatmaps of log2(FPKM + 1) gene expression data were clustered using Euclidean distance on Spearman correlations of the top 100 most variable genes using Ward’s squared linkage. GSEA analysis was performed using the Broad GSEA application^28^ on ranked lists by log2 fold changes from testing contrasts using DESeq2. *P* values were adjusted for multiple testing using the Benjamini-Hochberg procedure.

### Statistical analysis

Data were analyzed with Prism 9 software (GraphPad) and expressed as mean ± standard error (SE). Differences were analyzed for significance with Mann–Whitney test or Kruskal–Wallis one-way ANOVA for two groups. ANOVA was used to compare among multiple groups. All statistical tests were two-sided. *P* values less than 0.05 were considered statistically significant.

### Reporting summary

Further information on research design is available in the Nature Research Reporting Summary linked to this article.

### Supplementary information

Supplementary Information

Supplementary Data 1

Supplementary Data 2

Reporting Summary

## Data Availability

All RNAseq data are available in GEO (accession number GSE266932).
