# Supplementary material for: Pure estrogen receptor antagonists potentiate capecitabine activity in ESR1-mutant breast cancer
Source: NPJ Breast Cancer. 2024 Jun 8;10:42. doi: 10.1038/s41523-024-00647-1 (PMC11162492; doi:10.1038/s41523-024-00647-1)
Supplement: Supplementary file 1 — Supplementary Information [file 41523_2024_647_MOESM1_ESM.pdf]

SUPPLEMENTARY MATERIAL

Pure Estrogen Receptor Antagonists Potentiate Capecitabine Activity in *ESR1*-mutant Breast Cancer

Supplementary Figures: 5

Supplementary Data Files: 2

Supplementary Figure 1. IC50 studies performed on **(a)** MCF7 cells expressing either WT ER or the doxycycline induced Y537S ER mutation, or **(b)** *TP53* mutant T47D cells expressing WT ER or the doxycycline induced Y537S mutation, treated with 5FU, doxorubicin or paclitaxel (PTX). **(c)** Quantification of the colony confluency of control or p53 knock-out MCF7 cells expressing WT ER or the doxycycline inducible Y537S mutation, following treatment with vehicle, 5FU or fulvestrant as single agents or fulvestrant in combination with 5FU. Data are shown as mean ± SE. *** $P<0.001$. KO: knock out

a

MCF7

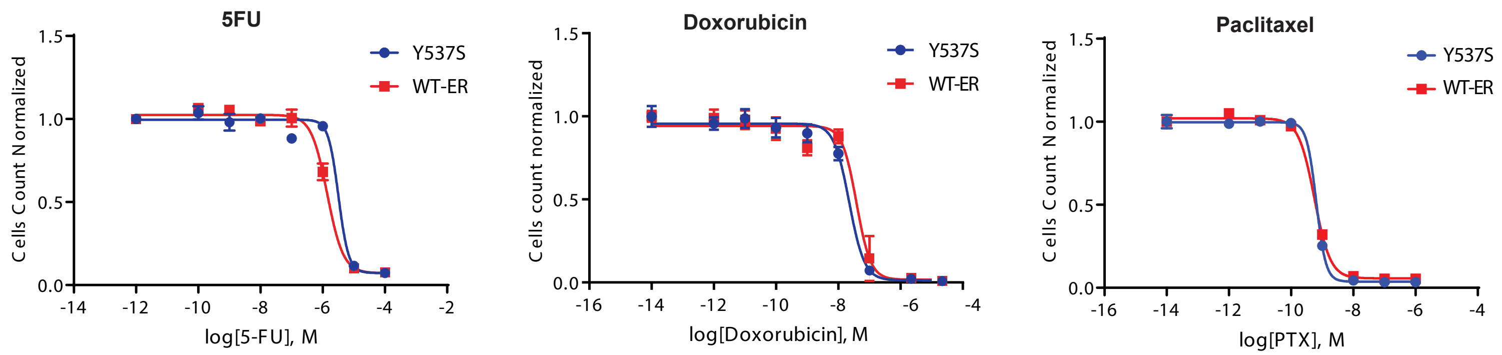

	Y537S	WT-ER
IC50	3.219e-006	1.402e-006

	Y537S	WT-ER
IC50	2.238e-008	3.844e-008

	Y537S	WT-ER
IC50	6.451e-010	5.623e-010

b

T47D

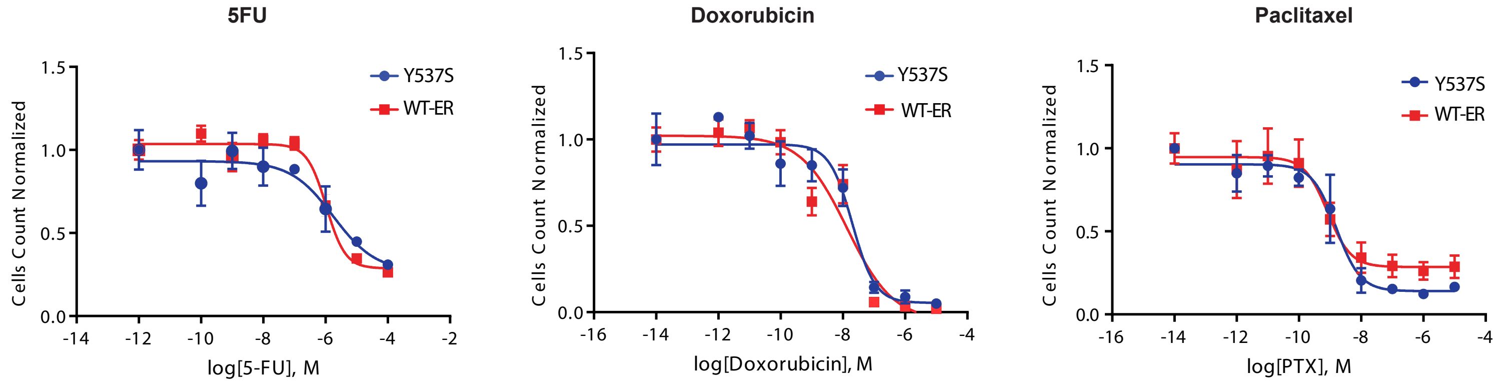

	Y537S	WT-ER
IC50	1.779e-006	1.053e-006

	Y537S	WT-ER
IC50	2.019e-008	1.368e-008

	Y537S	WT-ER
IC50	1.581e-009	8.317e-010

c

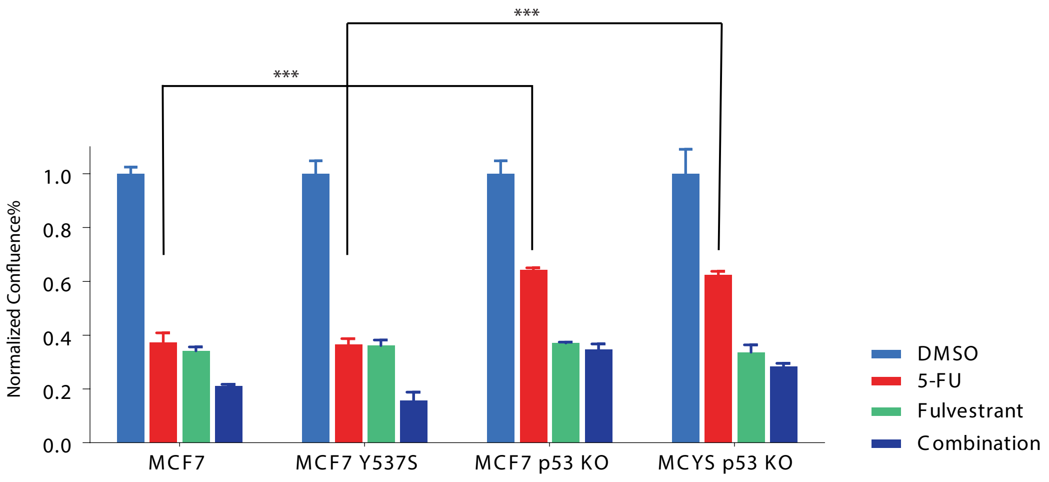

Supplementary Figure 2. (a-b) Number of downregulated (blue bars) and upregulated (green bars) genes in MCF7 cells without **(a)** or with **(b)** the expression of the Y537S mutation treated with 5FU, fulvestrant, or 5FU in combination with fulvestrant compared to vehicle treatment at different absolute log2FC (|FC| > 0, |FC| > 0.5 or |FC| > 1) and FDR<0.05 thresholds. ER: estrogen receptor; WT: wild type; FC: fold change; FDR: false discovery rate.

a

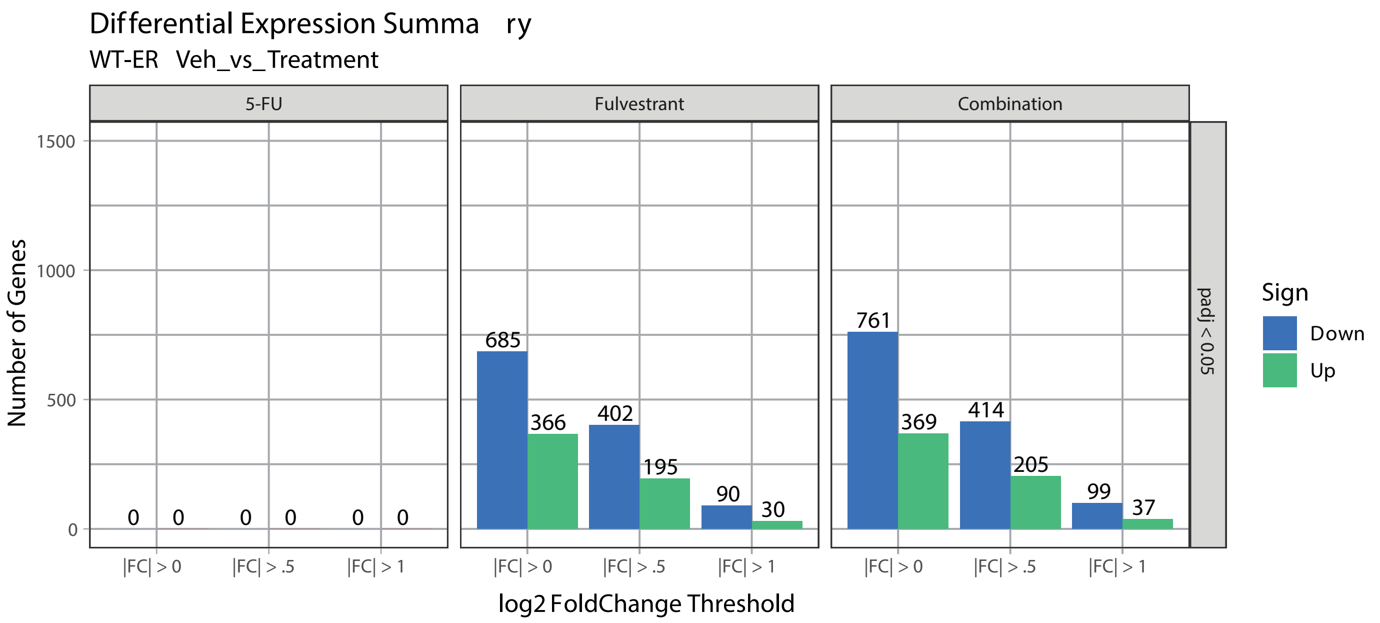

Differential Expression Summa ry
WT-ER Veh_vs_Treatment
5-FU
Fulvestrant
Combination
Number of Genes
1500
1000
500
0
0 0 0 0 0 0
685 366 402 195 90 30
761 369 414 205 99 37
|FC| > 0
|FC| > .5
|FC| > 1
log2 FoldChange Threshold
padj < 0.05
Sign
Down
Up

b

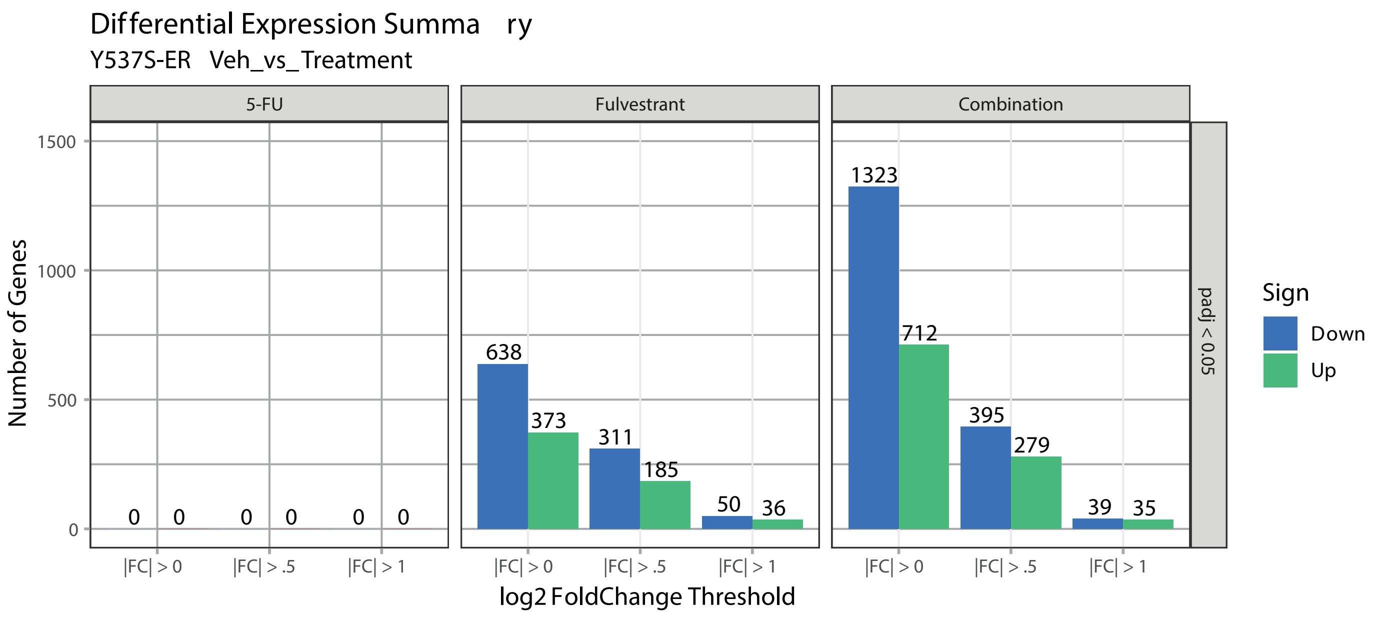

Differential Expression Summa ry
Y537S-ER Veh_vs_Treatment
5-FU
Fulvestrant
Combination
Number of Genes
1500
1000
500
0
0 0 0 0 0 0
638 373 311 185 50 36
1323 712 395 279 39 35
|FC| > 0
|FC| > .5
|FC| > 1
log2 FoldChange Threshold
padj < 0.05
Sign
Down
Up

Supplementary Figure 3. (a). *ESR1* mutant gene signature for MCF7 cells expressing WT ER treated with vehicle (Veh), 5FU, fulvestrant (Fulv), or 5FU in combination with fulvestrant (Com) showing relative expression values across samples (row centered and scaled log2(FPKM+1) expressions) with rows and columns unclustered. *ESR1* mutant gene signature consists of the top 100 genes by log2 fold change up in Y537S mutant vehicle treated cells compared to WT vehicle treated cells. **(b-c)** Venn diagrams showing the number of genes upregulated in MCF7 cells without **(b)** or with **(c)** the expression of Y537S mutation treated with fulvestrant or 5FU in combination with fulvestrant compared to vehicle treated cells for log2FC>=0.5 and FDR<=0.05 filtering thresholds. **(d)** Venn diagram showing the number of genes upregulated in MCF7 cells treated with 5FU in combination with fulvestrant compared to vehicle treated cells between cells without (WT) or with (Y537S ER) expression of the Y537S mutant for log2FC>=0.5 and FDR<=0.05 filtering thresholds. ER: estrogen receptor; WT: wild-type; FC: fold change; FDR: false discovery rate.

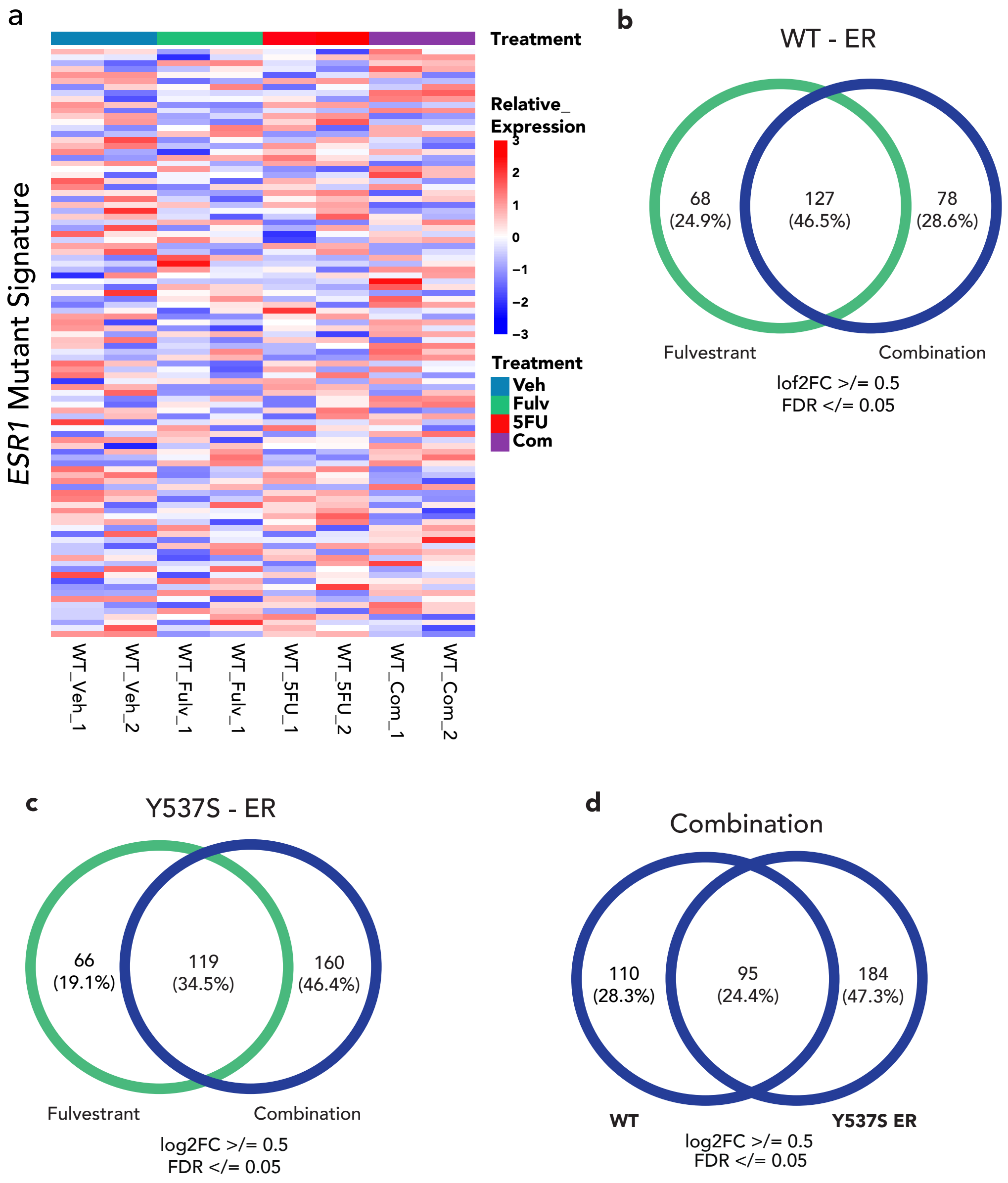
a
Treatment
Relative_
Expression
3
2
1
0
−1
−2
−3
Treatment
Veh
Fulv
5FU
Com
ESR1 Mutant Signature
WT_Veh_1
WT_Veh_2
WT_Fulv_1
WT_Fulv_1
WT_5FU_1
WT_5FU_2
WT_Com_1
WT_Com_2
b
WT - ER
68
(24.9%)
127
(46.5%)
78
(28.6%)
Fulvestrant
Combination
lof2FC >/= 0.5
FDR </= 0.05
c
Y537S - ER
66
(19.1%)
119
(34.5%)
160
(46.4%)
Fulvestrant
Combination
log2FC >/= 0.5
FDR </= 0.05
d
Combination
110
(28.3%)
95
(24.4%)
184
(47.3%)
WT
Y537S ER
log2FC >/= 0.5
FDR </= 0.05

Supplementary Figure 4. (a) The arrested fraction of cells at day 10 and day 28 in the Y537S ER mutant (1526) patient derived xenograft model. **(b-c)** Number of downregulated (blue bars) and upregulated (green bars) genes in PDX tumors expressing the Y537S mutation treated with 10 days (b) and 28 days **(c)** of capecitabine (Cap), fulvestrant, or capecitabine in combination with fulvestrant compared to vehicle treatment at different absolute log2 fold change (|FC| > 0, |FC| > 0.5 or |FC| > 1) and FDR<0.05 thresholds. PDX: patient-derived xenograft; ER: estrogen receptor; WT: wild-type; FC: fold change; FDR: false discovery rate.

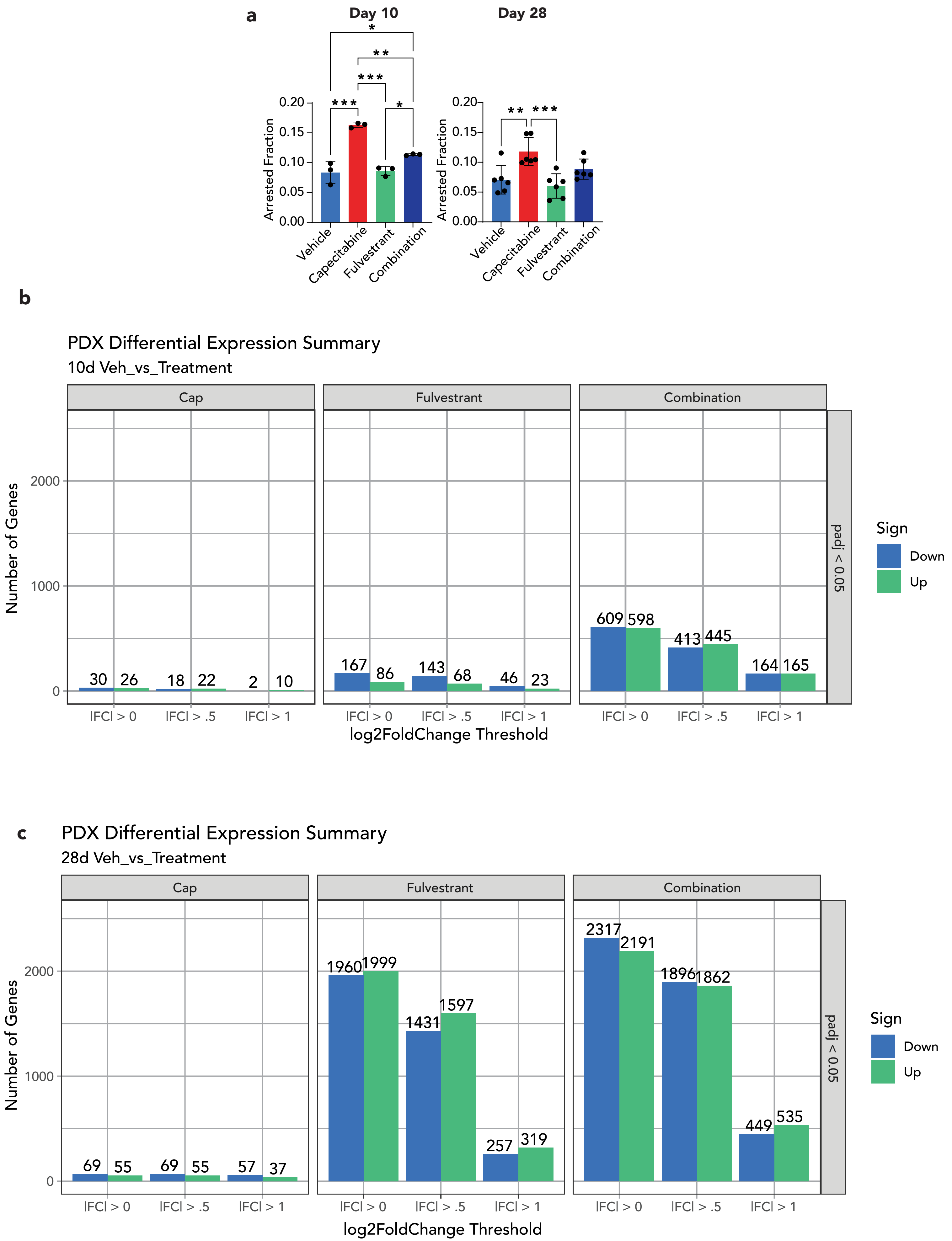
a
Day 10
Day 28
Arrested Fraction
0.20
0.15
0.10
0.05
0.00
*
**
***
*
**
***
Vehicle
Capecitabine
Fulvestrant
Combination
b
PDX Differential Expression Summary
10d Veh_vs_Treatment
Cap
Fulvestrant
Combination
Number of Genes
2000
1000
0
30 26 18 22 2 10
167 86 143 68 46 23
609 598 413 445 164 165
|FC| > 0
|FC| > .5
|FC| > 1
log2FoldChange Threshold
padj < 0.05
Sign
Down
Up
c
PDX Differential Expression Summary
28d Veh_vs_Treatment
Cap
Fulvestrant
Combination
Number of Genes
2000
1000
0
69 55 69 55 57 37
1960 1999 1431 1597 257 319
2317 2191 1896 1862 449 535
|FC| > 0
|FC| > .5
|FC| > 1
log2FoldChange Threshold
padj < 0.05
Sign
Down
Up

Supplementary Figure 5. Western blotting of p53 **(a)** and p21 **(b)** after p53 silencing in MCF7 cell with wild-type ER or Y537S ER mutation. **(c)** GAPDH as house-keeping protein. **(d)** molecular weight markers. WT: wild-type; ER: estrogen receptor.

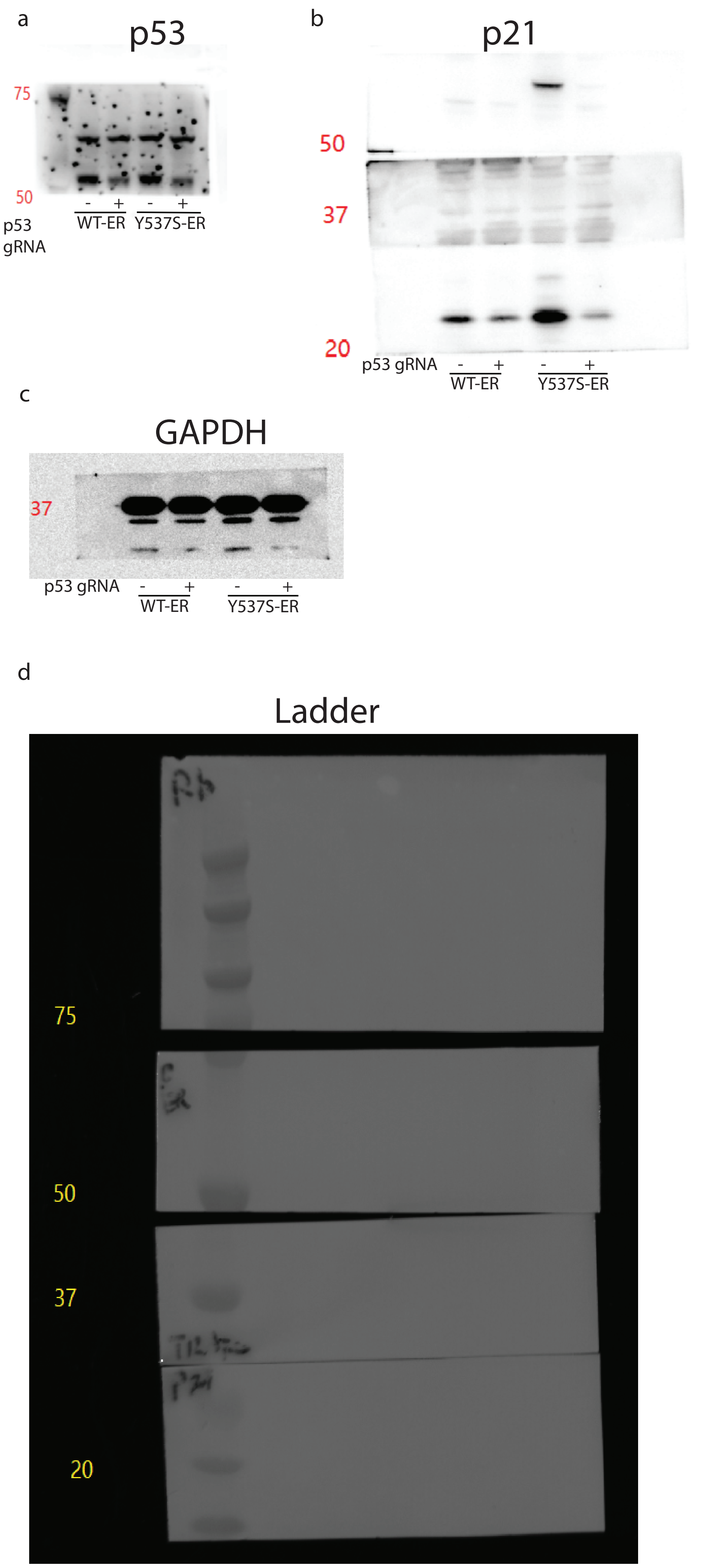
a
p53
75
50
p53 gRNA
- + - +
WT-ER
Y537S-ER
b
p21
50
37
20
p53 gRNA
- + - +
WT-ER
Y537S-ER
c
GAPDH
37
p53 gRNA
- + - +
WT-ER
Y537S-ER
d
Ladder
75
50
37
20

Supplementary Data 1. List of upregulated genes for fulvestrant and capecitabine treated.

See excel file.

Supplementary Data 2. List of antibodies used for Cyclic immunofluorescence.

See excel file.
